# Syndrome of Inappropriate Antidiuretic Hormone Secretion as the Initial Manifestation of Guillain-Barré Syndrome: A Case Report

**DOI:** 10.7759/cureus.108980

**Published:** 2026-05-16

**Authors:** Hema C Aremanda, Edgardo Torres Garcia, Nirupama Vemuri

**Affiliations:** 1 Internal Medicine, Sierra View Medical Center, Porterville, USA; 2 Nephrology, Sierra View Medical Center, Porterville, USA

**Keywords:** acute flaccid paralysis, albuminocytologic dissociation, ascending weakness, autonomic dysfunction, euvolemic hyponatremia, guillain-barré syndrome, hyponatremia, intravenous immunoglobulin, respiratory muscle weakness, siadh

## Abstract

Guillain-Barré syndrome is an acute immune-mediated polyradiculoneuropathy that commonly presents with progressive weakness and areflexia. This report describes euvolemic hypotonic hyponatremia preceding neuromuscular decline in a patient ultimately diagnosed with Guillain-Barré syndrome.

We report the case of a 69-year-old man who presented with generalized weakness and was found to have persistent euvolemic hypotonic hyponatremia. Initial evaluation suggested syndrome of inappropriate antidiuretic hormone secretion, and management with fluid restriction and sodium supplementation was initiated. Over the subsequent days, the patient developed rapidly progressive ascending weakness, areflexia, dysphagia, and respiratory failure. Cerebrospinal fluid analysis revealed elevated protein with mild pleocytosis and negative infectious studies, supporting the diagnosis of Guillain-Barré syndrome. Despite treatment with intravenous immunoglobulin, the patient required mechanical ventilation and transfer for plasmapheresis. With intensive supportive care and rehabilitation, gradual neurological recovery was observed over a six-month period. This case highlights the value of considering neurological etiologies in patients presenting with unexplained euvolemic hyponatremia and suggests that early electrolyte abnormalities may precede the development of overt neuromuscular disease.

## Introduction

Guillain-Barré syndrome (GBS) is an acute immune-mediated polyradiculoneuropathy and remains one of the most common causes of acute flaccid paralysis worldwide. It is typically characterized by rapidly progressive, symmetrical limb weakness, diminished or absent deep tendon reflexes, and variable sensory and autonomic involvement [1,2]. Although motor weakness is the hallmark feature, the clinical presentation can be heterogeneous, and early manifestations may be subtle, occasionally contributing to diagnostic delay [3].

Autonomic dysfunction is a well-recognized and clinically significant component of GBS, reported in a substantial proportion of patients. Clinical manifestations include cardiac arrhythmias, blood pressure lability, gastrointestinal dysmotility, urinary retention, and disturbances in fluid and electrolyte balance [4,5]. This dysregulation is thought to result from involvement of autonomic fibers and central regulatory pathways, including hypothalamic dysfunction and impaired baroreceptor signaling.

Hyponatremia is an important metabolic abnormality observed in GBS, reported in approximately 20%-50% of cases, and is most commonly attributed to the syndrome of inappropriate antidiuretic hormone secretion (SIADH) [6-8]. SIADH is characterized by non-osmotic release of antidiuretic hormone (ADH), leading to impaired free water excretion and dilutional hyponatremia. In GBS, this process is thought to be mediated by autonomic and neuroendocrine dysfunction, as well as inflammatory cytokine-induced stimulation of ADH release [6]. Recent studies and clinical guidelines have also recognized hyponatremia as a manifestation of autonomic involvement in GBS.

Although hyponatremia typically develops during the established course of GBS, emerging evidence suggests that autonomic and electrolyte disturbances may rarely precede the onset of classical neurological deficits. In such cases, hyponatremia is often initially attributed to systemic illness or medication effects, potentially delaying recognition of the underlying neurological disorder [7,8].

We present a case of GBS in which persistent euvolemic hypotonic hyponatremia due to SIADH preceded the development of classical neuromuscular manifestations. This case highlights the importance of considering underlying neurological disease in patients with unexplained and progressive hyponatremia and underscores the role of early autonomic involvement in atypical presentations.

## Case presentation

A 69-year-old Hispanic man with a history of type 2 diabetes mellitus (on metformin), hypertension (on amlodipine), and hyperlipidemia presented with several days of generalized weakness. On admission, vital signs were stable. Neurological examination revealed normal strength, intact sensation, and preserved deep tendon reflexes, with no focal deficits.

Initial laboratory evaluation demonstrated significant hyponatremia (serum sodium 123 mmol/L; corrected sodium 127 mmol/L in the setting of hyperglycemia with serum glucose 365 mg/dL), mild acute kidney injury, and microcytic anemia (hemoglobin 8.6 g/dL, mean corpuscular volume 72 fL) (Table 1). These findings confirmed true hypotonic hyponatremia, excluding pseudohyponatremia related to hyperglycemia. Given the severity of hyponatremia and associated renal dysfunction, the patient was admitted for further evaluation and close monitoring and was initially treated with isotonic intravenous fluids.

**Table 1:** Admission laboratory findings.

Parameter	Result	Reference range	Units
Hemoglobin	8.6	13.5-17.5	g/dL
Mean corpuscular volume (MCV)	72	80-100	fL
White blood cell count	6.8	4.0-11.0	×10⁹/L
Platelet count	310	150-400	×10⁹/L
Serum sodium	123	135-145	mmol/L
Corrected sodium	127	135-145	mmol/L
Serum potassium	4.2	3.5-5.0	mmol/L
Serum chloride	92	98-107	mmol/L
Serum bicarbonate	22	22-28	mmol/L
Blood urea nitrogen	28	7-20	mg/dL
Serum creatinine	1.4	0.6-1.3	mg/dL
Serum glucose	365	70-140	mg/dL
Serum albumin	4.1	3.5-5.0	g/dL

On hospital day two, renal function improved, with normalization of serum creatinine following initial isotonic intravenous fluid administration. Serum sodium demonstrated a transient improvement from 123 to 128 mmol/L prior to recognition of SIADH. During this period, hemoglobin declined from 8.6 to 7.0 g/dL without evidence of overt bleeding. Stool occult blood testing was negative, and upper gastrointestinal endoscopy revealed no evidence of active bleeding, ulceration, or malignancy. Platelet counts and liver function tests were within normal limits, with no evidence of hemolysis.

The decline in hemoglobin was most consistent with a dilutional process in the setting of intravenous fluid administration and evolving SIADH physiology, superimposed on an underlying microcytic anemia. There was no clinical evidence of hypovolemia to suggest hypovolemia-induced ADH secretion. Later that day, the patient developed new-onset lower extremity weakness with difficulty ambulating. A non-contrast computed tomography scan of the head showed no acute intracranial abnormalities. Serum sodium at that time was 125 mmol/L.

Given persistent hyponatremia despite initial management, further diagnostic evaluation was undertaken. Serum osmolality was low (265 mOsm/kg), while urine osmolality was inappropriately elevated at 476 mOsm/kg, indicating impaired free water excretion. Urinalysis was unremarkable. Urine sodium (140 mmol/L) and urine chloride (114 mmol/L) were elevated in the setting of clinical euvolemia. Thyroid-stimulating hormone and morning cortisol (16.6 µg/dL) were within normal limits, excluding hypothyroidism and adrenal insufficiency. Serum albumin was normal, and corrected sodium excluded pseudohyponatremia (Table 2).

**Table 2:** Laboratory evaluation of hyponatremia at the time of SIADH assessment. SIADH: syndrome of inappropriate antidiuretic hormone secretion

Parameter	Result	Reference range	Units
Serum sodium	125	135-145	mmol/L
Serum osmolality	265	275-295	mOsm/kg
Urine sodium	140	<30	mmol/L
Urine chloride	114	-	mmol/L
Urine osmolality	476	50-1,200	mOsm/kg
Serum creatinine	0.9	0.6-1.3	mg/dL
Serum glucose	180	70-140	mg/dL
Serum cortisol	16.6	5-25	µg/dL
Thyroid-stimulating hormone	2.1	0.4-4.5	mIU/L

The patient was not receiving diuretics or other medications known to induce hyponatremia, and clinical assessment demonstrated euvolemia, making alternative etiologies unlikely. These findings were diagnostic of euvolemic hypotonic hyponatremia consistent with SIADH. Fluid restriction (approximately 1-1.5 L/day) and oral sodium supplementation were initiated.

Over the subsequent two days, neurological status progressively worsened. By hospital day five, weakness had ascended to involve the upper extremities and was associated with dyspnea. Examination revealed generalized hypotonia, symmetric weakness, and absent deep tendon reflexes. During this period, serum sodium remained low, fluctuating between 125 and 127 mmol/L.

On hospital day six, the patient developed dysphagia and worsening respiratory muscle weakness, with a negative inspiratory force of −25 cm H₂O. Lumbar puncture demonstrated albuminocytologic dissociation, with elevated cerebrospinal fluid protein and minimal pleocytosis and negative infectious studies, consistent with GBS (Table 3).

**Table 3:** Cerebrospinal fluid analysis.

Parameter	Result	Reference range	Units
Appearance	Clear, colorless	-	-
White blood cells	19	0-5	cells/mm³
Red blood cells	40	0	cells/mm³
Protein	95	15-45	mg/dL
Glucose	75	45-80	mg/dL
Albumin	49.1	10-30	mg/dL
IgG index	0.52	<0.7	-
Oligoclonal bands	Negative	Negative	-
Myelin basic protein	<2	<4	ng/mL
Infectious studies	Negative	-	-

Electrodiagnostic testing (nerve conduction studies and electromyography) was not performed due to rapid neurological deterioration and the need for urgent respiratory support. The patient required transfer to the intensive care unit for close respiratory monitoring and subsequently developed respiratory failure requiring endotracheal intubation. Intravenous immunoglobulin (IVIG) therapy was initiated; however, neurological decline continued with progression to near-complete quadriplegia and severe bulbar dysfunction. No laboratory or clinical evidence of IVIG-associated hemolysis was observed.

**Figure 1:**
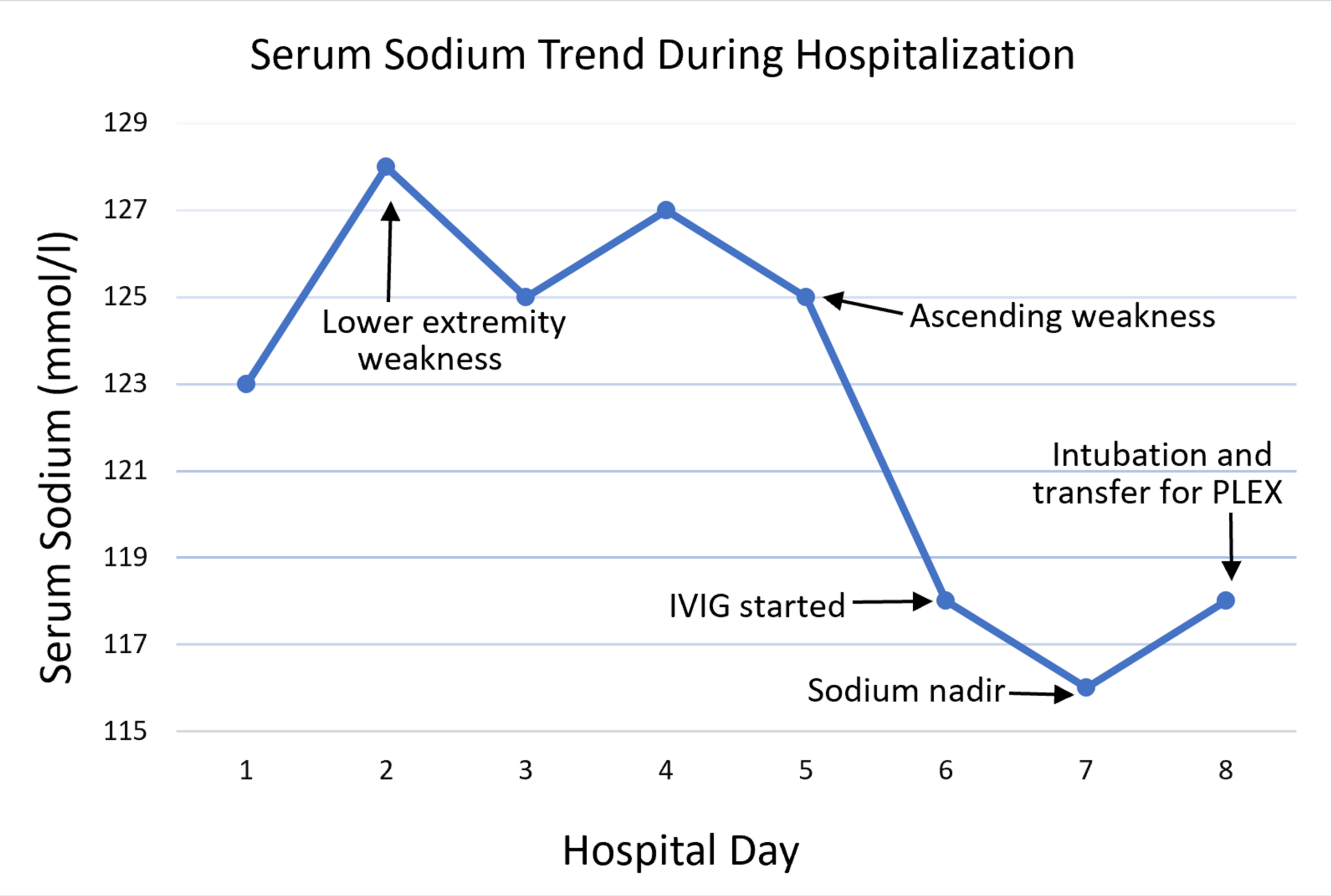
Serum sodium trend during hospitalization. The figure demonstrates persistent and refractory hyponatremia despite supportive measures, fluid restriction, sodium supplementation, and hypertonic saline therapy, preceding and paralleling neurological deterioration and respiratory failure. IVIG: intravenous immunoglobulin; PLEX: plasmapheresis

Concurrently, serum sodium declined further, reaching 118 mmol/L on hospital day six and a nadir of 116 mmol/L on hospital day seven, paralleling neurological deterioration. Hypertonic saline (3% sodium chloride) was initiated on hospital day eight for symptomatic and refractory hyponatremia, with careful monitoring to ensure gradual correction and avoid osmotic demyelination. Overall, the sodium trend demonstrated persistent and progressive hyponatremia preceding and paralleling neurological deterioration (Figure 1).

Following completion of IVIG without significant improvement, the patient was transferred to a tertiary care center for plasmapheresis, where he underwent six sessions. A tracheostomy was performed due to prolonged ventilatory dependence. With intensive rehabilitation, gradual neurological recovery was observed over a six-month period.

## Discussion

GBS is an acute immune-mediated polyradiculoneuropathy characterized by rapidly progressive weakness with variable involvement of sensory and autonomic fibers [1,2]. Although motor deficits predominate, autonomic dysfunction is common and contributes significantly to morbidity, manifesting as cardiovascular instability, gastrointestinal dysmotility, urinary retention, and disturbances in sodium and water homeostasis [3,4].

Hyponatremia is a recognized metabolic complication of GBS and is most often attributed to SIADH [6]. In this case, SIADH was supported by hypotonic hyponatremia (serum osmolality 265 mOsm/kg), inappropriately elevated urine osmolality (476 mOsm/kg), high urine sodium and chloride levels, clinical euvolemia, and normal thyroid and adrenal function (cortisol 16.6 µg/dL). Alternative causes-including hypovolemia, adrenal insufficiency, hypothyroidism, pseudohyponatremia, and medication-induced SIADH-were systematically excluded, strengthening the diagnostic inference.

Autonomic dysfunction in GBS can disrupt hypothalamic and baroreceptor-mediated regulation of ADH, resulting in inappropriate ADH secretion and impaired free water excretion. This neuroendocrine disturbance may occur early in the disease course, leading to hyponatremia prior to the onset of overt neurological deficits.

Consistent with this mechanism, hyponatremia in this patient preceded the onset of objective neurological deficits and progressively worsened in parallel with neurological deterioration, reaching a nadir during respiratory compromise. While this temporal relationship does not establish causality, it supports the concept that hyponatremia may reflect early autonomic involvement rather than being solely a consequence of critical illness.

Potential confounders were considered. Intravenous fluid administration and transfusion-related volume shifts may contribute to dilutional changes; however, hyponatremia was present prior to transfusion and persisted despite subsequent interventions. Although IVIG-associated pseudohyponatremia has been described, the presence of true hypotonic hyponatremia with inappropriately concentrated urine argues against this mechanism. Overall, the biochemical profile remained most consistent with SIADH. The decline in hemoglobin was most consistent with a dilutional process related to fluid administration and SIADH, superimposed on underlying microcytic anemia, and did not contribute to the neurological presentation.

These findings are consistent with prior reports demonstrating hyponatremia in approximately 20%-50% of patients with GBS and its association with increased disease severity, need for mechanical ventilation, and prolonged hospitalization [9,10]. Additional studies have further supported the association between hyponatremia and disease severity in GBS [11-13]. Recent European Academy of Neurology/Peripheral Nerve Society (EAN/PNS) 2023 guidelines also recognize autonomic dysfunction, including disturbances in fluid and electrolyte balance, as an important feature of GBS [14]. Early-onset SIADH preceding neurological deficits, as observed in this case, has been described but remains relatively uncommon [7,8].

Cerebrospinal fluid findings of albuminocytologic dissociation further supported the diagnosis [1,2]. Mild pleocytosis, as seen in this case, may occur early and does not exclude GBS when infectious causes are ruled out.

Overall, this case highlights SIADH as a potential early manifestation of GBS. Persistent, unexplained euvolemic hypotonic hyponatremia, particularly when progressive, may warrant consideration of underlying neurological disease and closer monitoring for impending neuromuscular and respiratory deterioration.

## Conclusions

This case highlights autonomic dysfunction as an early manifestation of GBS. Persistent euvolemic hyponatremia consistent with SIADH preceded the onset of classical neurological deficits and paralleled disease progression. Although this temporal relationship does not establish causality, this single observational case supports the association between electrolyte disturbances, early autonomic involvement, and severe disease manifestations, including respiratory compromise. In patients with otherwise unexplained or progressive hyponatremia, particularly in the setting of evolving weakness, consideration of an underlying neurological process may facilitate earlier diagnosis, closer respiratory monitoring, and timely management of GBS.
